# Southern Dental Association

**Published:** 1885-05

**Authors:** 


### SOUTHERN DENTAL ASSOCIATION.

### SEVENTEENTH ANNUAL MEETING, AT NEW ORLEANS.

REPORTED FOR THE INDEPENDENT PRACTITIONER, BY MRS. M. W. J.

The annual meeting of the Southern Dental Association, for the year 1885, was held in New Orleans, commencing Tuesday, March 31st, and lasting four days.

The sessions were held at Tulane Hall, where, fifteen years ago, the second annual meeting was held. This hall is located in what was then known as the Mechanics' Institute. It now forms one of the group of buildings known as Tulane University, the old University of Louisiana.

Through the liberality of the faculty, with Col. Wm. Preston Johnston at its head, the free use of the hall and committee rooms was given, both night and day, to the Southern Dental Association, and also to the National Board of Dental Examiners.

Although southern in name, this Association is national in character, embracing in its membership, and inviting to the privileges of the floor, members of the profession from all parts of the country.

Among the representative men present and participating in the discussions may be mentioned Drs. McKellops Morrison and Spalding of St. Louis, Taft and H. A. Smith of Cincinnati, Harlan and Cushing of Chicago, Robinson of Michigan, Patrick of Illinois, Bonwill of Philadelphia, Parmly Brown of New York, Coyle of Maryland, Morgan of Tennessee, Rawls of Kentucky, Teague and Wright of South Carolina, Catching and Wardlaw of Georgia, Chisholm of Alabama, and many others. The Association brought together probably three hundred dentists, from all parts of the country.

The officers were—

*President*—A. O. Rawls, Lexington, Ky.

*First Vice-President*—W. R. Clifton, Waco, Tex.

*Second Vice-President*—T. S. Waters, Baltimore, Md.

*Third Vice-President*—B. H. Catching, Atlanta, Ga.

*Recording Secretary*—R. A. Holliday, Atlanta, Ga.

*Corresponding Secretary*—J. L. Fountain, Bryan, Tex.

*Treasurer*—H. A. Lowrance, Athens, Ga.

*Chairman Committee of Arrangements*—J. R. Walker, New Orleans.

The morning session of the first day was occupied by the usual formalities of organization, a brief but graceful address of welcome by Dr. L. A. Thurber, responded to by Dr. Morgan, of Nashville, and the President's annual address, in which was incorporated an able paper on Pyorrhœa Alveolaris.

The discussion of this paper was postponed until the subject of Pathology and Therapeutics was reached, in the regular order of business.

The Association then adjourned, and a meeting of the National Board of Examiners was held, with representatives from the State Boards of Illinois, Indiana, Michigan, Ohio, Georgia, Maryland, Mississippi and Louisiana.

During the afternoon session Dr. Wardlaw, of Augusta, Ga., read a scholarly paper on Dental Hygiene, and also a valuable paper on the same subject by Dr. B. F. Arrington, of Goldsboro, N. C. The former is briefly summarized as follows:

Dr. Wardlaw spoke of the continual struggle between health and disease, due to the perishable nature of our physical organs, efforts towards physical perfection resulting in the formulation of the science of hygiene. The laws of dental hygiene take a wide range, going back prior to the marriage state, in the choice of mates. But such laws are nearly always a dead letter, men being governed by their passions, regardless of posterity. Spartan laws aimed at "the survival of the fittest." Medical science of to-day fosters the perpetuation of the most undesirable progenitors.

The most practical thing to be done in dental hygiene, is to impress upon mothers the general principle that "clean teeth will not decay." If the mother commences with the first tooth of her first babe, a foundation is laid for good habits, and the care of the teeth will become necessary to comfort. The position was taken that inorganic mineral elements are not assimilated. Phosphatic food may be good for tonic effects, but it is not prophylactic. He gave as practical instructions the use of a moderately stiff brush, and the quill toothpick, and a soluble ant-acid powder. He condemned pastes and soap, as likely to remain too long in contact with the gums. The teeth should be exercised on good food, and on chewing-gum. Tobacco chewing prevents deposits of tartar.

Dr. Arrington's paper was also devoted to common sense and practical suggestions, rather than to theories. He considered the field limited to the care of the teeth and tissues, and the placing of them in condition for comfort and usefulness. Outside of that we get beyond our specialty. Dieting, with a view to its influence on future generations, is absurd and unpractical. Commence the care of the teeth at the age of nine or ten. Let the treatment be temporizing, not attempting too much for young patients. Age, locality and structure of tooth, should decide as to the proper material for filling. Pyorrhœa alveolaris should be called by the common name of scurvy, and treated with local applications of sulphuric acid, for months, if necessary. Prescriptions of mountain air, sea air, etc., are absurd, as far as teeth are concerned.

These papers elicited a lengthy and spirited discussion. All secret preparations were condemned. The dentist should have the exact formula of everything he prescribes or uses. Soft brushes were generally recommended, moderately stiff ones being injurious, and hard brushes grooving the enamel.

Dr. Teague advocated a brush with very long bristles, to be used with a sweeping motion, rather than scouring.

Dr. Walker said if 100 represented the range of brushes, 25 was the one to use. Soap was generally condemned as lubricating, where friction was required. The use of soap was approved by others, however, as dissolving oily deposits from greasy food.

Instruction of the masses as to the importance of preserving deciduous teeth, and in proper methods of cleaning the teeth, was urged. Tooth washes were condemned. It is impossible to so compound a wash as that it shall benefit both teeth and gums; what is good for one must be bad for the other.

The question of the administration of lime-salts, whether animal or mineral, as food or medicine, as tonic or prophylactic, was argued at length, Drs. Morgan and Patrick supporting the position taken by the essayists, Drs. Taft, Spalding, Walker and others opposing, and maintaining the feasibility of benefiting coming generations through the mothers of yet unborn babes.

Dr. Walker attributed the insufficiency of ordinary food to supply the demands of the teeth, to its lack of lime-salts; Dr. Spalding to lack of assimilative power on the part of the patient, phosphatic medicinal preparations as stimulants to assimilation being recommended; Dr. Patrick attributed bad development to a lack of function, an increase of supplies consequently being useless, while Dr. Morgan maintained that a moderate supply of even the poorest article of food, instancing rice, contained a supply of mineral elements amply sufficient to more than rebuild the entire skeleton every seven years.

The soft teeth, so universal in the Mississippi Delta, were attributed by Dr. Walker to lack of mineral elements in the drinking water. This was ascribed by Dr. Morgan to the filthy condition of the city. He admitted, however, that children in Tennessee, amidst surroundings of squalor and want, had good teeth. Dr. Walker supported his position by reference to certain limestone districts in Texas, whose sanitary conditions were so bad that typhoid fever reigned, and yet the teeth were very fine. Drs. Taft, Spalding and others, gave illustrations and cited cases supporting the position of Dr. Walker.

Cleanliness, as preventive of decay, was generally agreed upon, Dr. Morgan taking exception, and attributing decay rather to vitiated fluids. He also considered alkaline fluids as special cause for alarm.

Drs. Spalding, Taft, Walker and others, attributed decay largely to lack of exercise of teeth and jaw in the proper mastication of proper food. The use of chewing gum and tobacco for exercising the jaws, as advocated in the paper of Dr. Wardlaw, they do not consider an efficient nor sufficient substitute for normal exercise in mastication, and vicious as inducing an undue secretion of saliva. Dr. Taft also attributed decay to the use of food which was irritating to the mucous membrane of the alimentary tract, producing undue stimulation of the glands and consequent mixed secretions of the mouth, injurious to the teeth. The injurious effects of mouth-breathing were discussed by Drs. Taft, Rawls, Spalding, Morgan and others, the vitiated condition of the fluids and the foul breath of the mouth-breather being attributed to evaporation of watery particles, and the rapid chemical changes which take place in saliva when exposed to atmospheric currents. This was sustained by Dr. Patrick, who said that "internal could not be converted into external membrane without creating unhealthy tissues."

Temporizing work was unanimously condemned. The most perfect work possible should be done in every case, the age, locality and structure of each tooth determining what should be considered best in each instance. Dr. Morrison attributed decay to too long intervals between meals, the consequent inactivity of the jaws promoting disintegration.

(TO BE CONTINUED.)

